# Atypical Presentation of Acute Myocardial Infarction As Syncope and Abdominal Pain in the Absence of Chest Pain or Discomfort

**DOI:** 10.7759/cureus.99892

**Published:** 2025-12-22

**Authors:** Sakher S Ja'anini, Aseel J Al-Ma'ai'a

**Affiliations:** 1 General Medicine, Al-Mahabba Hospital, Madaba, JOR

**Keywords:** abdominal pain, acute st-elevation myocardial infarction, atypical presentation, heart failure, syncope

## Abstract

Atypical presentations of acute myocardial infarction can make early diagnosis challenging, particularly when chest pain or discomfort is absent. We present a case of a 60-year-old man with diabetes, hypertension, a previous myocardial infarction (MI), and heart failure with reduced ejection fraction who presented with syncope and severe abdominal pain but no chest pain or discomfort. The ECG demonstrated an inferior ST-segment elevation myocardial infarction. He received dual antiplatelet therapy and anticoagulation before emergent transfer for coronary angiography, which revealed right coronary artery occlusion.

‎We concluded that acute myocardial infarction can present without chest pain or discomfort and instead manifest with syncope and/or abdominal pain, especially in patients with risk factors for atypical presentations. Clinicians should maintain a high index of suspicion for acute coronary syndrome in such cases to avoid delays in diagnosis and reperfusion therapy, as delays may increase morbidity and mortality.

## Introduction

Myocardial infarction (MI) manifests clinically as part of acute coronary syndromes (ACS), a potentially life-threatening condition [1]. It typically results from the rupture of an atherosclerotic coronary plaque, followed by thrombosis and obstruction of distal perfusion, ultimately leading to myocardial ischemia and necrosis [2,3]. Risk factors include a mix of non-modifiable factors such as older age, male sex, family history, and genetic predisposition, and modifiable factors including dyslipidemia, hypertension, smoking, diabetes, obesity, inactivity, and poor diet [2,3].

Acute myocardial infarction is most commonly characterized by retrosternal chest pain described as dull, squeezing, or pressure-like, radiating to the left arm or jaw, and usually accompanied by sweating, nausea, or shortness of breath [2,4]. Nevertheless, a subset of patients may present with atypical symptoms that make early recognition challenging [5]. Epigastric or generalized abdominal pain, transient loss of consciousness (syncope), back pain, diaphoresis, or even silent MI can be the initial manifestations, particularly among elderly patients, those with diabetes mellitus and/or a history of coronary artery disease [1,4-7]. Moreover, sex differences can influence how myocardial infarction presents [4]. Such unusual presentations may lead to delayed diagnosis and treatment, thereby increasing the risk of complications [1].

‎‎We present a case of acute myocardial infarction that initially manifested with a brief episode of syncope and generalized abdominal pain, emphasizing the importance of considering cardiac causes even in patients without typical chest pain or discomfort.

## Case presentation

A 60-year-old male with a known history of type 2 diabetes mellitus, essential hypertension, previous myocardial infarction that led him to the ICU in a coma for about 10 days, and heart failure with reduced ejection fraction (HFrEF), presented to the Emergency Department at Al Mahabba Hospital, Madaba, Jordan, following an episode of syncope. The exact details of his prior medical history were unavailable as previous records could not be retrieved. According to family members, the loss of consciousness lasted only a few seconds, and the patient regained consciousness spontaneously but could not recall the event. He complained of severe abdominal pain prior to arrival and during presentation but denied chest pain before, during, or after the syncopal episode. 

‎On examination, the patient was alert and oriented with mild confusion, as he took time to answer questions and to process information. He appeared ill, diaphoretic, and in pain, holding his abdomen with both hands. He appeared overweight. His vital signs were: heart rate 63 BPM, blood pressure 76/50 mmHg, and oxygen saturation 94% on room air. Neurological exam showed no focal neurological weaknesses. Chest examination revealed good bilateral air entry without wheezes or crackles, and cardiovascular examination showed distant heart sounds without jugular venous distension or lower-limb edema. The abdomen was diffusely tender, most pronounced in the epigastric and upper quadrants but without rigidity. Left periorbital swelling and bruising were noted secondary to the fall. The patient has been a chronic smoker using vape for many years. 

**Figure 1:**
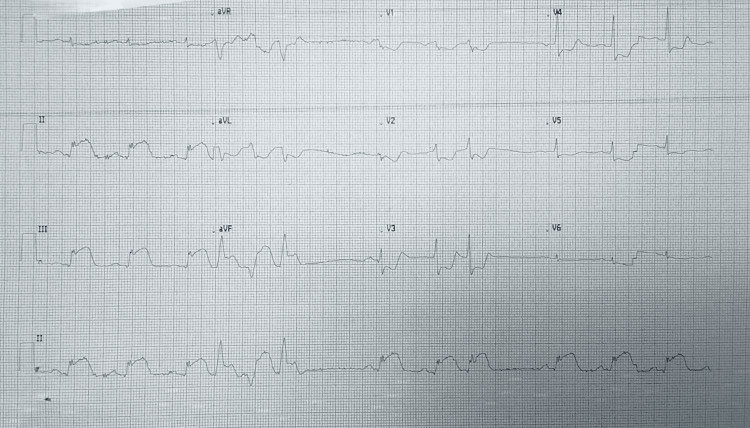
ECG at the ER of the first tertiary hospital, showing ST-segment elevation in leads II, III, and aVF with reciprocal depression in leads I and aVL, as well as ST-segment depression in leads V1–V5.

‎Electrocardiogram (ECG) demonstrated ST-segment elevation in leads II, III, and aVF with reciprocal depression in leads I and aVL, as well as ST-segment depression in leads V1-V5, consistent with a massive inferior ST-elevation myocardial infarction (STEMI) (Figure 1). Laboratory investigations, including kidney function test, liver function tests, amylase, troponin I, D-dimer, and complete blood count, were unremarkable, with negative cardiac enzymes likely due to early presentation (Table 1). His home medications included bisoprolol 5 mg once daily, rosuvastatin 20 mg once daily, aspirin 100 mg once daily, clopidogrel 75 mg once daily, metformin 850 mg twice daily, and valsartan 160 mg twice daily. 

**Table 1:** Lab results at the first presentation in the ER of Al-Mahabba Hospital. Hgb: haemoglobin, HCT: haematocrit, RBC: red blood cell count, WBC: white blood cell count, MCV: mean corpuscular volume, MCH: mean corpuscular haemoglobin, MCHC: mean corpuscular haemoglobin concentration, PLT: platelet count, RDW-CV: red cell distribution width–coefficient of variation, ALT: alanine aminotransferase, AST: aspartate aminotransferase, SGOT: serum glutamic oxaloacetic transaminase, SGPT: serum glutamic pyruvic transaminase.

Lab Results	Results	Normal Range
Hgb	13.8	13.5 - 17.5 (g/dl)
Hct	40.5	37 - 53 (%)
RBC	4.58	4.5 - 5.9 (x10^6 µL)
WBC	11.3	4.5 - 11 (x10^3 µL)
MCV	88.428	80 - 100 (fl)
MCH	30.131	26 - 34 (pg)
MCHC	34.074	32 - 36 (%)
PLT	156	150 - 450 (x10^3 µL)
RDW-CV	14.6	11.5 - 13.1 (%)
Neutrophil (NEU)	68	35 - 66 (%)
Lymphocytes (LYM)	24	24 - 44 (%)
Monocyte (MON)	8	4 - 10 (%)
Urea	26	12.8 - 42.8 (mg/dL)
Creatinine	0.94	0.70 - 1.20 (mg/dL)
Sodium	141	135 - 148 (mmol/L)
Potassium	4.26	3.5 - 5.0 (mmol/L)
Chloride	108	97 - 107 (mmol/L)
AST (SGOT)	17	Up to 40 (U/L)
ALT (SGPT)	20	Up to 41 (U/L)
Alkaline Phosphatase (ALP)	90	40 - 115 (U/L)
Bilirubin -Total	0.45	< 1.20 (mg/dL)
Bilirubin - Direct	0.17	Up to 0.40 (mg/dL)
Amylase	115	28 - 80 U/L
Troponin I	Negative	Negative
D- Dimer	Negative	< 0.5

‎In the Emergency Department, he received aspirin 300 mg po (chewable) re-loading, clopidogrel 300 mg (orally) re-loading, unfractionated heparin 5000 IU (intravenously), normal saline 0.9% 500 mL bolus, and paracetamol 1 g (intravenously). A cardiology consultation was obtained, and the catheterization laboratory at a nearby tertiary (50 minutes away) hospital was activated. The patient was transferred emergently for coronary angiography and further management. At the time of transfer, he was hemodynamically stable with blood pressure 89/60 mmHg, heart rate 71 bpm, and oxygen saturation 96% on room air.

**Figure 2:**
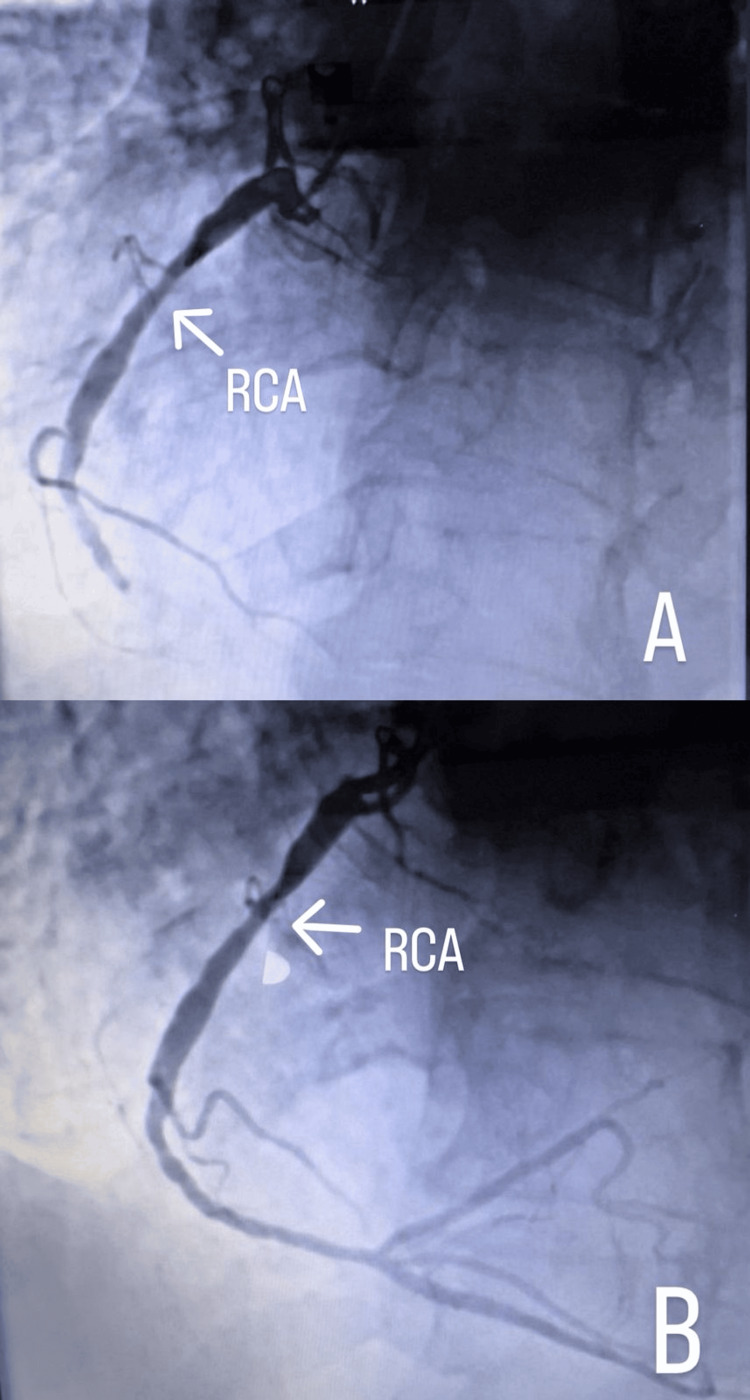
Coronary angiography A: Showing cut-off distal contrast flow in the right coronary artery (RCA) due to total arterial occlusion (pre-stenting). B: Showing improved distal contrast flow in the RCA (post stenting).

**Figure 3:**
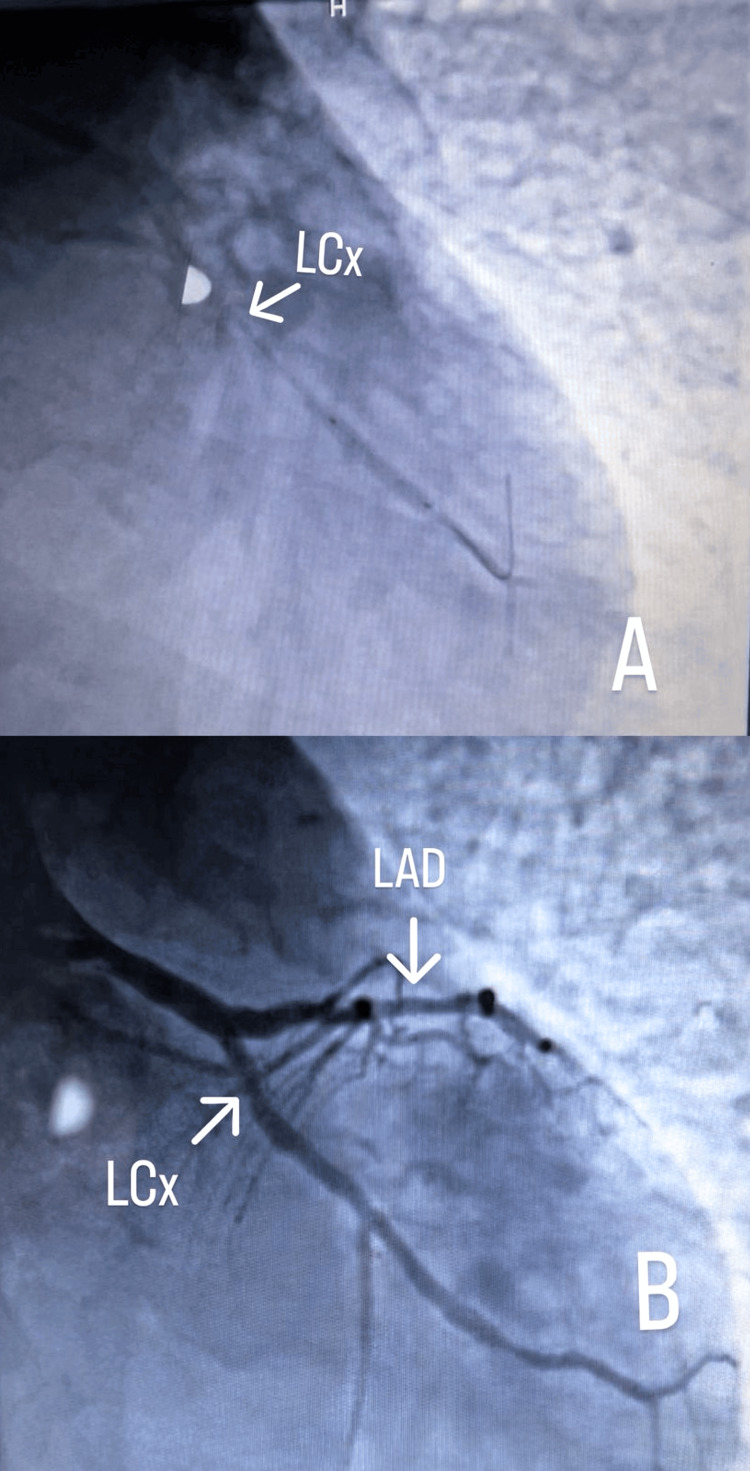
Coronary angiography A: Showing left circumflex artery (LCx) during stenting (note the guide wire). B: Showing LCx contrast flow post stenting; left anterior descending artery (LAD) can also be noted above it.

At the tertiary hospital, coronary angiography revealed a distal total occlusion of the right coronary artery (RCA) (Figure 2). The left main coronary artery (LMCA) was long and normal. The left circumflex artery (LCx) demonstrated a patent previous stent with an additional distal lesion, while the left anterior descending artery (LAD) showed an 80% stenotic lesion (Figure 3). The patient was right-dominant, and two drug-eluting stents (DES) were successfully deployed - one in the RCA and the other in the LCx. Percutaneous coronary intervention (PCI) to the left main coronary artery was planned for later.

**Figure 4:**
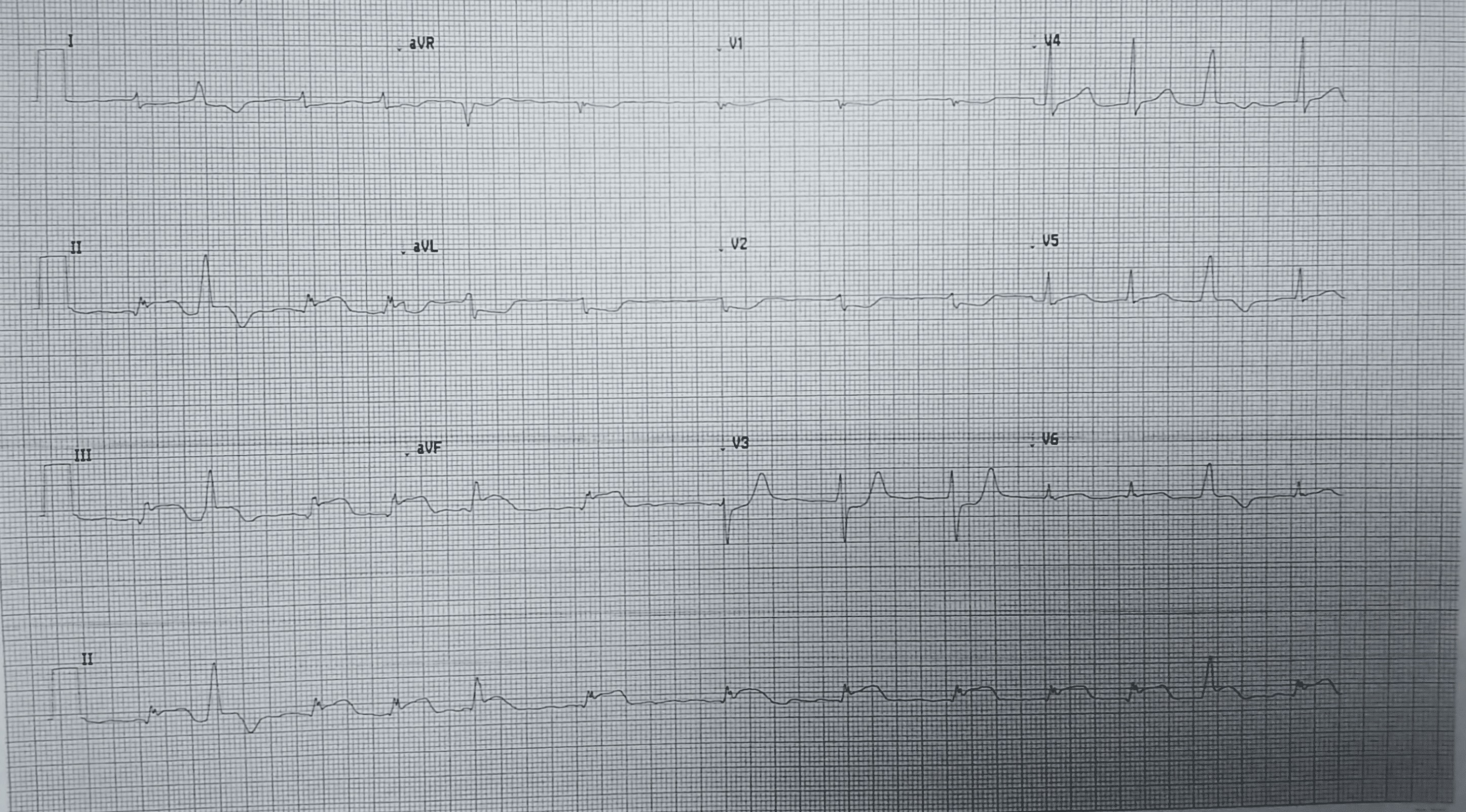
ECG post the first two drug eluting stents placement in the first tertiary hospital showing marked improvement in ST segments.

**Figure 5:**
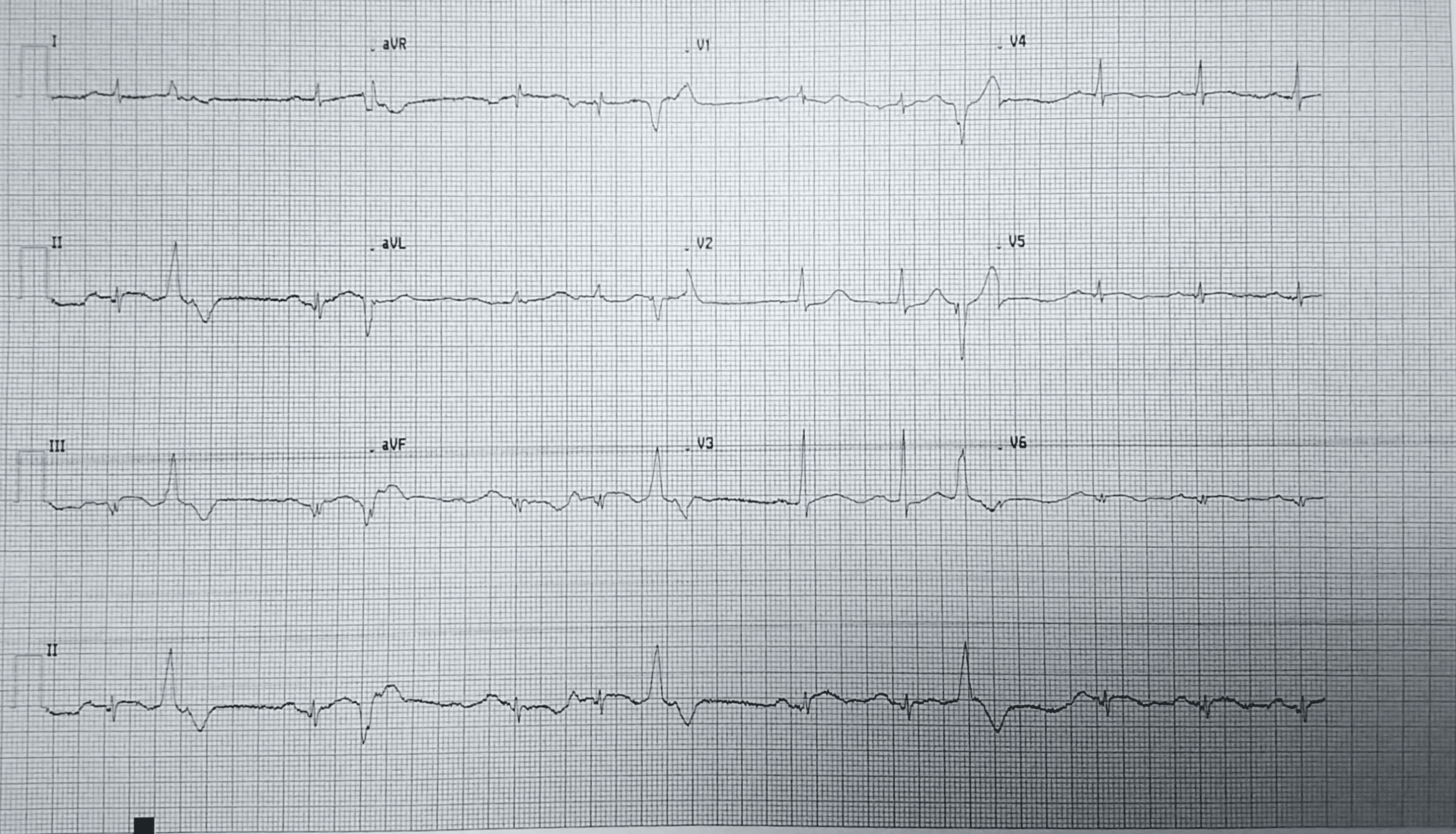
ECG done one day after stenting at the first tertiary hospital, showing further improvement of ST segments, low voltage QRS complexes, and multiple PVCs. PVC: premature ventricular contraction.

Post-stenting ECG showed marked improvement in the ST segments (Figure 4). ECG one day after stenting showed further improvement in the ST segments (Figure 5). He was monitored in the coronary care unit for three days and subsequently discharged in stable condition. Post-procedure echocardiography demonstrated an ejection fraction of 30%, mild mitral and tricuspid regurgitation, a pulmonary artery pressure of 25 mmHg, and no pericardial effusion. A non-contrast cranial and cervical CT scan performed during hospitalization revealed left subcutaneous periorbital edema without orbital fractures, intact globes, and non-displaced fractures of the right anterior and posterior tubercles of the C3 and C4 transverse foramina. A left temporal arachnoid cyst was also identified, for which neurosurgical follow-up was advised.

‎One week later, the patient underwent elective coronary angiography at another tertiary hospital, which demonstrated 80% stenosis of the left anterior descending artery (LAD) with all previously placed stents remaining patent. Three additional drug-eluting stents were successfully deployed in the LAD. He was admitted for one day of observation and subsequently discharged in stable condition. On discharge, he appeared well, with no abdominal pain, jaundice, pallor, or peripheral edema. There was no jugular venous distension, good bilateral air entry, and distant heart sounds. His latest echocardiogram showed an ejection fraction of 25%. An intracardiac defibrillator device (ICD) was not implanted. His discharge medications included clopidogrel 75 mg once daily, aspirin 100 mg once daily, bisoprolol 2.5 mg once daily, furosemide 40 mg once daily, metformin 850 mg twice daily, and rosuvastatin 20 mg once daily. Due to the patient's low blood pressure (100/70 mmHg), valsartan was titrated to 40 mg twice daily. Blood pressure monitoring and follow-up were planned to uptitrate valsartan to the target dose in subsequent follow-ups. Dapagliflozin 10 mg once daily was also added to his regimen in his follow-ups.

**Figure 6:**
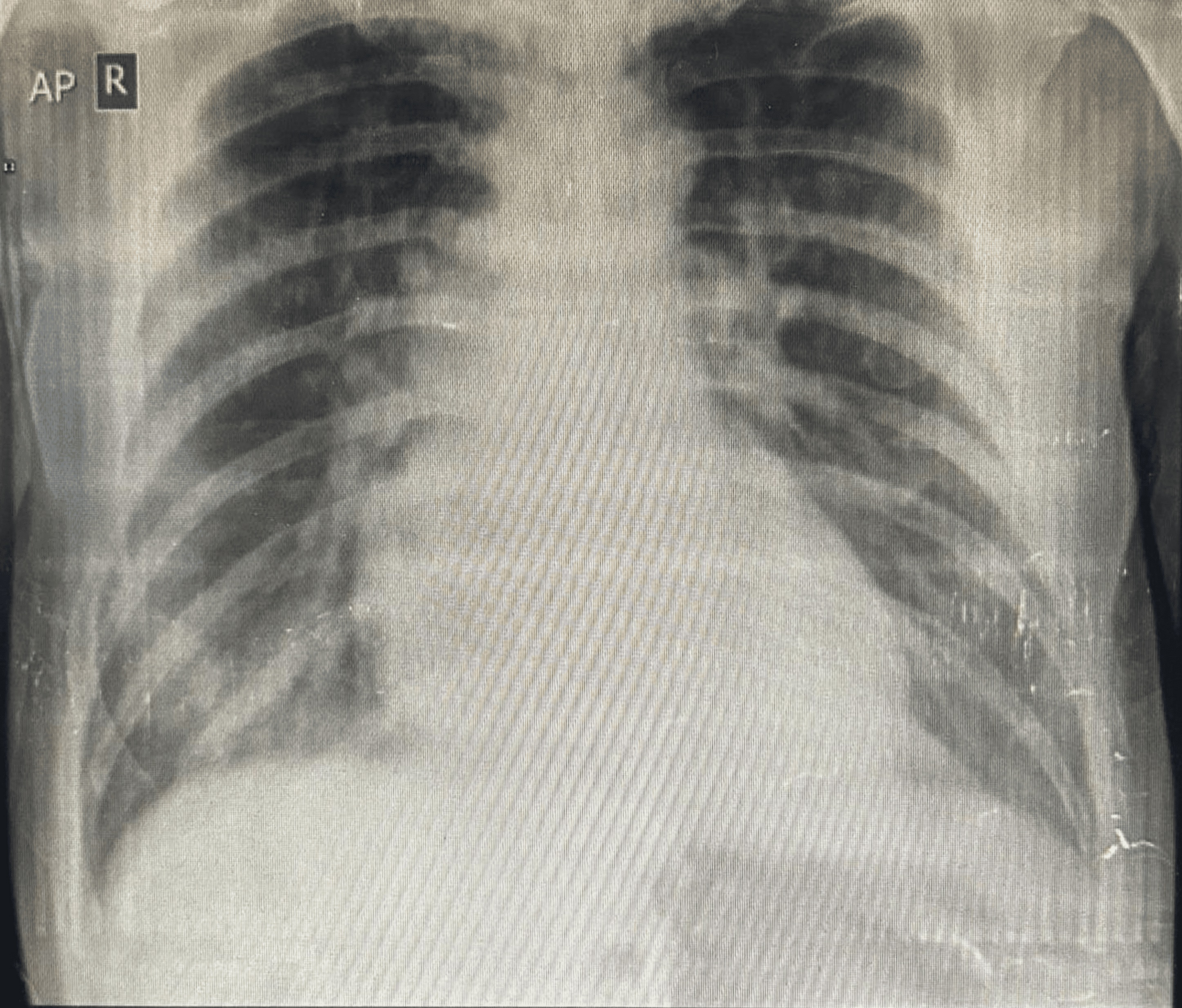
Portable chest X-ray at the second presentation in the ER of Al-Mahabba Hospital, showing cardiomegaly and bilateral hilar prominence suggesting pulmonary venous congestion.

‎One month after his first presentation, the patient returned to the Emergency Department at Al Mahabba Hospital complaining of shortness of breath, i.e., "feeling tired and out of breath." His blood pressure was 110/70 mmHg, oxygen saturation 87%, pulse 85 bpm, and temperature 36.8°C. he was diaphoretic and tachypneic. Examination revealed good bilateral air entry with bibasilar fine crackles, a distended tympanic abdomen, and distant heart sounds without jugular venous distension or lower-limb edema. It was noted that he had been noncompliant with his furosemide therapy. Laboratory investigations, including kidney function test, complete blood count, troponin I, and D-dimer, were unremarkable (Table 2). ECG showed low-voltage QRS complexes with premature ventricular contractions, unchanged from his post-catheterization tracing. Chest X-ray revealed cardiomegaly and bilateral hilar prominence, suggesting pulmonary venous congestion (Figure 6). He was treated with intravenous furosemide 40 mg and oxygen supplementation. His symptoms improved within an hour, and his saturation returned 94% on room air. He was advised to remain compliant with his medications and follow up with his cardiologist as soon as possible. 

**Table 2:** Lab results at the second presentation in the ER of Al-Mahabba Hospital Hgb: haemoglobin, Hct: haematocrit, RBC: red blood cell count, WBC: white blood cell count, MCV: mean corpuscular volume, MCH: mean corpuscular haemoglobin, MCHC: mean corpuscular haemoglobin concentration, PLT: platelet count, RDW-CV: red cell distribution width–coefficient of variation.

Lab Results	Results	Normal Range
Hgb	13.5	13.5 - 17.5 (g/dl)
Hct	41.8	37 - 53 (%)
RBC	4.60	4.5 - 5.9 (x10^6 µL)
WBC		4.5 - 11 (x10^3 µL)
MCV	90.87	80 - 100 (fl)
MCH	29.348	26 - 34 (pg)
MCHC	32.297	32 - 36 (%)
PLT	139	150 - 450 (x10^3 µL)
RDW-CV	13.6	11.5 - 13.1 (%)
Neutrophil (NEU)	65	35 - 66 (%)
Lymphocytes (LYM)	27	24 - 44 (%)
Monocyte (MON)	8	4 - 10 (%)
Urea	22	12.8 - 42.8 (mg/dL)
Creatinine	0.73	0.70 - 1.20 (mg/dL)
Sodium	139	135 - 148 (mmol/L)
Potassium	4.33	3.5 - 5.0 (mmol/L)
Chloride	104	97 - 107 (mmol/L)
Troponin I	Negative	Negative
D-Dimer	Negative	<0.5

‎One week later, the patient presented again with severe shortness of breath. He was cyanosed, diaphoretic, tachypneic, and irritable. On arrival, his blood pressure was 110/75 mmHg, temperature 37.1°C, pulse 120 bpm, and oxygen saturation 67%. Examination revealed bibasilar crackles and distant heart sounds. Shortly after presentation, he developed cardiopulmonary arrest. Cardiopulmonary resuscitation was initiated and continued for one hour without return of spontaneous circulation, and death was declared. From our understanding and after talking to his wife, the patient was not compliant with all his medications, especially the heart failure medications and follow-up appointments.

## Discussion

As mentioned earlier, typically MI presents as retrosternal chest pain that is dull, squeezing, or pressure-like, often radiating to the left arm or jaw. This pain is frequently accompanied by diaphoresis, nausea, and shortness of breath [2,4].

‎The mechanism of MI-related pain can be explained by the nervous pathway of visceral pain of the myocardium injury, as the afferent sympathetic neurons travel from the myocardium to the cardiac plexus and then to the spinal cord without synapsing [8]. These neurons predominantly connect to lamina I in the dorsal horn, with additional connections to lamina V [8]. Somatic nociceptive fibers also synapse with lamina I neurons, potentially causing 'crosstalk' between somatic and visceral pathways, leading to referred pain [8]. Extensive connections between cardiac sympathetic plexi, ganglion chain, and spinal cord explain the widespread expression of myocardial infarction pain in the upper body [8].

‎Atypical presentations may arise when these pain pathways are altered. Contributing factors include diabetic neuropathy, prior coronary artery disease, age-related reduction in pain perception due to ischemic changes in sensory nerves and cortical processing, and autonomic nervous system dysfunction [1,6,7]. Men are generally reported in many studies to have a higher likelihood of typical myocardial infarction presentations [4]. However, a study by Ferry et al. showed the opposite, finding that men were more likely than women to present with atypical symptoms [4]. Our patient fit several of these criteria: he was male, he had diabetes mellitus, a prior MI, a 10-day history of coma raising concern for cortical ischemic changes, and was in his sixties, making an atypical presentation more likely. He exhibited multiple cardiovascular risk factors, including hypertension, diabetes mellitus, advanced age, male gender, active smoking, and excess body weight. All should put acute coronary syndrome as a differential diagnosis for his syncope and abdominal pain, even in the absence of chest pain or discomfort.

‎Syncope is defined as a sudden, transient loss of consciousness and postural tone resulting from temporary global cerebral hypoperfusion. When cardiac in origin, insufficient cerebral blood flow occurs because the heart fails to generate adequate cardiac output, which may result from ischemic structural damage or electrical abnormalities following myocardial infarction [9]. Inferior myocardial infarction, in particular, may present with abdominal pain, nausea, and syncope due to vagally mediated responses and overlapping visceral afferent pathways [8,10].

‎Management of inferior ST-elevation MI (STEMI) begins with rapid ABC (Airway, Breathing, Circulation) assessment to ensure airway patency, adequate breathing, and stable circulation. Oxygen is administered only if saturation is below 90%, and the patient is monitored for complications such as bradycardia, hypotension, or right ventricular involvement [2,11,12]. After stabilization, a 12-lead ECG and, when indicated, a right-sided ECG are obtained [2,12]. Initial therapy includes aspirin loading, P2Y12 inhibitor loading, and anticoagulation [2,12]. Avoid nitrates and provide volume using intravenous crystalloids to ensure adequate preload in the right ventricular infarction, as the right ventricle is preload dependent, and nitrates will decrease preload and precipitate hypotension [2,12]. Once inferior STEMI is confirmed, early reperfusion becomes the priority. Guidelines recommend immediate cath-lab activation for primary PCI, aiming for a door-to-balloon time of ≤90 minutes [13]. Rapid revascularization of the usually affected right coronary artery reduces infarct size and prevents complications [13]. If PCI cannot be performed within the recommended timeframe, fibrinolytic therapy is considered, although PCI remains the preferred strategy due to better outcomes [11].

‎Myocardial infarction (MI) can worsen heart failure (HF) through several pathways. Besides the initial loss of myocardium, additional structural problems such as myocardial rupture, ventricular septal defects, papillary muscle dysfunction, or free wall rupture can sharply increase cardiac stress, with septal rupture carrying the poorest prognosis [14]. Patients with prior infarction scars, pre-existing HF, or other cardiomyopathies are also more prone to deterioration, and systemic comorbidities like anemia, chronic kidney disease, or chronic lung disease can further impair cardiac function [14]. Chronic ischemia may promote myocardial fibrosis and stiffen the ventricle, contributing to long-term dysfunction [14].

Management centers on urgent correction of mechanical complications when present and initiation or optimization of pharmacotherapy, including angiotensin converting enzyme (ACE) inhibitors/angiotensin receptor blockers (ARBs)/angiotensin receptor-neprilysin inhibitors (ARNI), β-blockers, diuretics, mineralocorticoid receptor antagonists, and sodium-glucose co-transporter 2 (SGLT2) inhibitors [15,16]. Neurohormonal antagonists, including ACE inhibitors, ARBs, β-blockers, and aldosterone antagonists, inhibit or even reverse left ventricular remodeling [16]. Automated implantable cardioverter defibrillators (AICDs) and cardiac resynchronization therapy devices (CRTs) are beneficial in select patients with HF who are at risk for sudden cardiac death from ventricular tachyarrhythmias and who have worsening HF from cardiac dyssynchrony [15]. Revascularization and strategies that limit adverse remodeling are key to preventing further HF progression [15].

‎Sudden cardiac death is the leading cause of mortality in heart failure, occurring six to nine times more often than in the general population due to the high burden of ventricular arrhythmias and myocardial scarring [17]. Another major mode of death is progressive pump failure, marked by declining cardiac output, persistent congestion, and recurrent hospitalizations [18]. Other contributors include recurrent ischemic events, thromboembolism, and comorbidities such as renal dysfunction or infection [18]. Moreover, medication non-adherence was associated with an increased risk of all-cause mortality and cardiovascular hospitalizations in heart failure patients [19].

‎Our case emphasizes the importance of considering acute coronary syndrome as a differential diagnosis in patients with cardiovascular risk factors who present with syncope and/or abdominal pain, even in the absence of chest pain or discomfort, particularly given the possibility of atypical myocardial infarction presentations. Additionally, this case highlights the critical role of adherence to heart failure medications, regular follow-up visits, and patient education on the potential risks and adverse outcomes associated with poor compliance.

‎Limitations

‎The patient was treated in three unrelated hospitals, and records were obtained via email and phone, leading to possible gaps in documentation. Additionally, past medical records could not be retrieved, and we relied solely on verbal reports from his wife and daughter.

## Conclusions

Patients with acute myocardial infarction may present with syncope or abdominal pain in the absence of chest pain or discomfort. Several factors contribute to these atypical presentations, including older age, gender, diabetes mellitus, and a history of myocardial infarction or stroke. Therefore, acute coronary syndrome should remain an important consideration in patients who present with syncope and/or abdominal pain, particularly when they have cardiovascular risk factors or known predictors of atypical presentation. Failure to recognize these atypical symptoms can lead to delays in diagnosis and reperfusion therapy, resulting in larger infarct size, worsening heart failure, and increased morbidity and mortality. This case also underscores the importance of strict adherence to heart failure medications and regular follow-up visits, as both are strong predictors of clinical outcomes.
